# Comprehensive miRNome-Wide Profiling in a Neuronal Cell Model of Synucleinopathy Implies Involvement of Cell Cycle Genes

**DOI:** 10.3389/fcell.2021.561086

**Published:** 2021-03-04

**Authors:** Elisabeth Findeiss, Sigrid C. Schwarz, Valentin Evsyukov, Thomas W. Rösler, Matthias Höllerhage, Tasnim Chakroun, Niko-Petteri Nykänen, Yimin Shen, Wolfgang Wurst, Michael Kohl, Jörg Tost, Günter U. Höglinger

**Affiliations:** ^1^Department of Translational Neurodegeneration, German Center for Neurodegenerative Diseases, Munich, Germany; ^2^Department of Neurology, School of Medicine, Technical University of Munich, Munich, Germany; ^3^Department of Neurology, Hannover Medical School, Hanover, Germany; ^4^Department of Psychiatry, Washington University School of Medicine, St. Louis, MO, United States; ^5^Laboratory for Epigenetics and Environment, Center National de Recherche en Génomique Humaine, CEA–Institut de Biologie François Jacob, Evry, France; ^6^Institute of Developmental Genetics, Helmholtz Center Munich, Munich, Germany; ^7^Genome Engineering, German Center for Neurodegenerative Diseases (DZNE), Munich, Germany; ^8^Munich Cluster for Systems Neurology (SyNergy), Munich, Germany; ^9^Medizinisches Proteom-Center, Ruhr-University Bochum, Bochum, Germany

**Keywords:** Parkinson’s disease, alpha-synuclein, microRNA, next-generation sequencing, cell cycle, cyclin D

## Abstract

Growing evidence suggests that epigenetic mechanisms like microRNA-mediated transcriptional regulation contribute to the pathogenesis of parkinsonism. In order to study the influence of microRNAs (miRNAs), we analyzed the miRNome 2 days prior to major cell death in α-synuclein-overexpressing Lund human mesencephalic neurons, a well-established cell model of Parkinson’s disease (PD), by next-generation sequencing. The expression levels of 23 miRNAs were significantly altered in α-synuclein-overexpressing cells, 11 were down- and 12 upregulated (*P* < 0.01; non-adjusted). The *in silico* analysis of known target genes of these miRNAs was complemented by the inclusion of a transcriptome dataset (BeadChip) of the same cellular system, revealing the G0/G1 cell cycle transition to be markedly enriched. Out of 124 KEGG-annotated cell cycle genes, 15 were present in the miRNA target gene dataset and six G0/G1 cell cycle genes were found to be significantly altered upon α-synuclein overexpression, with five genes up- (*CCND1*, *CCND2*, and *CDK4* at *P* < 0.01; *E2F3*, *MYC* at *P* < 0.05) and one gene downregulated (*CDKN1C* at *P* < 0.001). Additionally, several of these altered genes are targeted by miRNAs hsa-miR-34a-5p and hsa-miR-34c-5p, which also modulate α-synuclein expression levels. Functional intervention by siRNA-mediated knockdown of the cell cycle gene cyclin D1 (*CCND1*) confirmed that silencing of cell cycle initiation is able to substantially reduce α-synuclein-mediated cytotoxicity. The present findings suggest that α-synuclein accumulation induces microRNA-mediated aberrant cell cycle activation in post-mitotic dopaminergic neurons. Thus, the mitotic cell cycle pathway at the level of miRNAs might offer interesting novel therapeutic targets for PD.

## Introduction

In recent years, epigenetic regulation by microRNAs (miRNAs) has been linked to the pathogenesis of Parkinson’s disease (PD) (Goodall et al., 2013; Singh and Sen, 2017; Goh et al., 2019; Ravanidis et al., 2020). PD is primarily characterized by the progressive loss of dopaminergic neurons in distinct midbrain regions. A neuropathological hallmark of PD is the formation of intraneuronal protein inclusions, predominantly comprising the protein α-synuclein (αSyn) (Spillantini et al., 1997; Lees et al., 2009), which is encoded by the *SNCA* gene on chromosome 4. There are different approaches to model synucleinopathies *in vitro*. We chose a robust PD model with adenoviral overexpression of human wild-type αSyn in post-mitotic Lund human mesencephalic (LUHMES) neurons (Lotharius et al., 2005; Höllerhage et al., 2014).

Epigenetic mechanisms such as miRNA-mediated regulation of gene expression have been suggested to be involved in the etiology of PD (Goh et al., 2019). miRNAs are endogenous single-stranded non-coding RNAs with a size of ∼23 nucleotides and known to play a key role as post-transcriptional regulators through binding to messenger RNAs (mRNA) (He and Hannon, 2004). The diverse roles of miRNAs have been extensively studied in the context of PD (Goodall et al., 2013; Singh and Sen, 2017). In fact, several PD-related processes, such as apoptosis and mitochondrial integrity, have been reported to be modulated by differential miRNA expression (Minones-Moyano et al., 2011; Wang et al., 2016). Furthermore, the study of *in vitro* and *in vivo* PD models revealed a link between several miRNAs and PD pathology, such as miR-7 and miR-153 (Je and Kim, 2017; Titze-de-Almeida and Titze-de-Almeida, 2018). Both have been shown to regulate *SNCA* mRNA and the αSyn protein levels in mouse models of PD (Junn et al., 2009; Doxakis, 2010). Additionally, the analysis of miRNA levels in PD patients revealed a clear dysregulation of several members of the miRNA family let-7, miR-92, and miR-184 in peripheral blood and in distinct brain regions, e.g., the substantia nigra (Martins et al., 2011; Briggs et al., 2015; Tatura et al., 2016). One single miRNA might regulate the expression of multiple target genes (Cai et al., 2009). Therefore, alterations of a few miRNAs can affect a multitude of genes, thus influencing PD pathology in multiple steps by targeting different pathways (Martinez and Peplow, 2017). It is currently unknown whether certain miRNAs are involved in biological compensation processes at an early stage of αSyn upregulation and could therefore be used as novel drug targets to attenuate synucleinopathies.

Adenoviral *SNCA* overexpression results in increased intracellular αSyn protein levels and ∼ 50% cytotoxicity levels at day 6 post transduction in differentiated LUHMES neurons (Höllerhage et al., 2014; Chakroun et al., 2020). In the present study, we performed a miRNome-wide screen in *SNCA*-overexpressing LUHMES neurons at day 4 post transduction and focused on (1) altered levels of miRNAs and their target genes and (2) identifying a functional involvement of dysregulated biological pathways. This time point was chosen to observe αSyn-mediated effects since it represents a phase in which the cells are challenged with a significant increase of intracellular αSyn levels, whereas cytotoxicity remains limited.

## Materials and Methods

### Cell Culture

Lund human mesencephalic cells were cultured as described previously (Höllerhage et al., 2017). Briefly, cells were plated in T75 flasks (EasYFlasks, Nunclon DELTA, Thermo Fisher Scientific, Waltham, MA, United States) coated with 50 μg/mL poly-L*-*ornithine (Sigma-Aldrich, St. Louis, MO, United States) in DMEM/F12 growth medium (Sigma-Aldrich) with 1% N2-supplement (Life Technologies, Carlsbad, CA, United States) and 0.04 μg/mL human basic fibroblast growth factor (bFGF; PeproTech, Rocky Hill, CT, United States). Multi-well dishes and flasks (Nunc MicroWell plates, Thermo Fisher Scientific, Waltham, MA, United States) were coated with 50 μg/mL poly-L-ornithine (Sigma-Aldrich) at 37°C overnight and washed three times with phosphate-buffered saline (PBS; LifeTechnologies) followed by coating with 5 μg/mL fibronectin (Sigma-Aldrich) for 24 h in the incubator (37°C, 5% CO_2_). For experiments, cells were plated at a density of 110,000 cells/cm^2^ in differentiation medium [DMEM/F12 with 1% N2-supplement, 1 μg/mL tetracycline, 0.49 mg/mL dibutyryl cyclic-AMP (Sigma-Aldrich), and 2 ng/mL human glial cell-derived neurotrophic factor (GDNF; R&D Systems, Minneapolis, MN, United States)]. Cells were routinely tested for mycoplasma contamination.

### Virus Transduction

Adenoviral vectors (AV) harboring the complementary DNA of human wild-type α-synuclein (*SNCA*) or green fluorescent protein (*GFP*) under cytomegalovirus (CMV) promoter and enhancer (BioFocus DPI, Leiden, Netherlands) were added at a multiplicity of infection (MOI) of two to LUHMES cells 48 h after differentiation started (Höllerhage et al., 2017). After 24 h, cells were washed three times with PBS to remove adenoviral particles. Fresh differentiation medium was supplemented and cells kept in culture until readout. The control cells were treated in the same manner without the addition of virus.

### Immunocytochemistry

Cells were plated on 8-well ibidi μ-slides (ibidi, Gräfelfing, Germany). After 8 days of differentiation, cells were fixated with 4% PFA, followed by blocking and permeabilization in 5% horse serum with 0.1% Triton X-100 in PBS. Then, the cells were incubated with primary antibodies [mouse monoclonal anti MAP2 (clone AP20, Millipore MAB3418); rabbit anti β-III-tubulin (clone 9F3, Cell signaling #2128)] for 2 h at room temperature and washed three times with PBS. Incubation with fluorescently labeled secondary antibodies [anti rabbit Alexa 594-conjugated (Thermo Scientific), anti mouse Alexa 488-conjugated (Thermo Scientific)], for 1 h, was followed by 4′,6-diamidino-2-phenylindole (DAPI) staining. Cells were incubated with 1 μg/mL DAPI in PBS for 5 min, and washed three times with PBS. Images were subsequently taken using an inverted microscope (DMI6000, Leica Microsystems) using a 40x objective and the corresponding Leica Software (Leica microsystems, Wetzlar).

### Total RNA and miRNA Isolation

On day 4 post transduction, the cells were washed with PBS once and detached mechanically. After spinning down (300 *g* for 5 min at 4°C) the biomaterial was briefly stored at −80°C until RNA was isolated. For total RNA extraction the RNeasy Plus Kit was used strictly according to manufacturer‘s protocol (Qiagen, Hilden, Germany). In brief, 350 μL of buffer RLT Plus were added to the collection tube containing the defrosted biomaterial and subsequently vortexed for 30 s. The transfer of the lysate to a gDNA eliminator spin column was followed by centrifugation for 30 s at 8,000 *g*. The flow-through was mixed with 350 μL of 70% ethanol (v/v) and transferred to an RNeasy spin column. After centrifugation for 15 s at 8,000 *g*, the column was washed by adding 700 μL of buffer RW1 to the column, followed by another centrifugation step for 15 s at 8,000 *g*. Thereafter, the column was washed twice with 500 μL of buffer RPE by centrifugation at 8,000 *g*, for 15 s and 2 min, respectively. After drying the membrane by centrifugation at full speed for 1 min, the column was placed into a new 1.5 mL collection tube. RNA was eluted by adding 30 μL of RNase-free water directly to the spin column membrane and centrifugation for 1 min at 8,000 *g*. For miRNA extraction, the miRNeasy and RNeasy MinElute Cleanup Kits were used according to the manufacturer‘s protocol (Qiagen) for enrichment of small RNAs from cultured cells. In brief, 700 μL QIAzol lysis reagent was added to the collection tube containing the defrosted biomaterial and homogenized by vortexing for 1 min. After incubation at room temperature for 5 min, 140 μL of chloroform were added and the lysate mixed by shaking the collection tube for 15 s, followed by incubation at room temperature for 3 min. Phase separation was accomplished by centrifugation for 15 min at 12,000 *g* at 4°C. 350 μL of the upper aqueous phase were transferred to a new reaction tube before and 525 μL of ethanol were added. After thorough mixing, 700 μL sample were transferred to an RNeasy mini column followed by centrifugation for 15 s at 8,000 *g*. This step was repeated with the remainder of the sample. The flow-through was transferred to a 2 mL collection tube and 500 μL of ethanol were added. Thereafter, the sample was transferred to an RNeasy MinElute spin column and centrifuged for 15 s at 8,000 *g*. This step was repeated with the remainder of the sample. The column was washed with 500 μL of buffer RPE by centrifugation for 15 s at 8,000 g and with 500 μL of 80% ethanol (v/v) by centrifugation for 2 min at 8,000 *g*. To dry the membrane, the column was centrifuged at full speed for 5 min. Then, the miRNA was eluted by adding 14 μL of RNase-free water to the spin column membrane followed by centrifugation for 1 min at full speed. The concentrations of the isolated miRNA and RNA of each condition were quantified using a NanoDrop spectrophotometer (Thermo Fisher). Thereafter, samples were stored at −80°C for further analysis.

### Transcriptome/mRNA Expression Analysis

Cells were cultured in 10 cm petri dishes until day 6 of differentiation. After washing the cells once with PBS, 500 mL RLT buffer (Qiagen, Hilden, Germany), activated with β-mercaptoethanol (Sigma-Aldrich) according to the manufacturer’s instructions, was added to the petri dishes. Total RNA of three replicates was collected in sterile cryotubes (Sarstedt, Nümbrecht, Germany) using a cell scraper (Carl Roth, Karlsruhe, Germany) and stored at −80°C until use. The expression analysis was performed with Illumina HumanHT-12_V3 bead chips (Illumina, San Diego, CA, United States). Data obtained from Illumina HumanHT-12_V3 bead chips (Illumina, San Diego, CA, United States) measurements was analyzed in order to detect differentially regulated transcripts. To this end, the output obtained from the Illumina GenomeStudio Software (v. 1.0.6) was used as input for further data preprocessing and differential analyses steps, which were implemented as workflow within the KNIME analytics platform (^1^ v3.6.2). However, since KNIME allows integration of R code, R capabilities (lumi v2.28.0, affy v1.54.0, genefilter v1.58.1, limma v3.32.10, lumiHumanAll.db v1.22.0, and lumiHumanIDMapping v1.10.1) were used. All R packages are available from Bioconductor^2^. We used the *lumi* package in order to carry out basic preprocessing of the input data. To this end, the *lumiExpresso* function of lumi was used with the following settings: For background correction the *forcePositive* method has been applied, *quantile normalization* was carried out and *log2 transformation* was used for variance stabilization. After data preprocessing, expression values (features) that did not show enough variation to allow reliable detection of differential expression have been removed. Using the *nsFilter* method of the R package *genefilter* the interquartile range (IQR) was used as a measure for dispersion and the 0.5 quantile of the IQR values has been used as cutoff for removal of unnecessary features. For each of the remaining features a two-tailed *t*-test was performed with the *rowttests* function from the *genefilter* package. In order to control the rate of type I errors, when conducting multiple *t*-tests, the Benjamini–Hochberg method was applied (FDR controlling). Features with adjusted *p* values < 0.05 (608 features) are considered differentially regulated and used for further analyses. As additional data processing steps several mappings procedures were carried out in order to annotate the data with additional information. The R packages *lumiHumanAll.db* and *lumiHumanIDMapping* were used to retrieve both gene symbols and unique Entrez Gene identifiers (GeneIDs).

### Next Generation Sequencing of Small RNAs

50 ng of small RNA enriched fractions were converted into barcoded cDNA libraries using the NEBNext Multiplex Small RNA Library Prep kit (New England BioLabs, Ipswich, MA, United States) for next-generation sequencing on the Illumina platform. The protocol was performed following the manufacturer’s instruction using the Caliper Sciclone liquid handler station (Perkin Elmer, Waltham, MA, United States). Total RNA or small RNA enriched fractions were ligated to the 3′ SR adaptor (1:4 dilution), hybridized to the Reverse Transcription Primer (1:4 dilution) and ligated to the 5′ SR adaptor (1:4 dilution). Reverse transcription was performed on the ligated RNA using ProtoScript II Reverse. cDNA libraries were indexed and amplified using the following conditions: denaturation for 30 s at 95°C, 16 amplification cycles – 15 s at 95°C, 30 s at 62°C, 15 s at 70°C–and a final extension for 5 min at 70°C. Libraries were purified using a QIAquick kit (Qiagen). Finally, the library size selection was performed using AMPure XP Beads (Beckman Coulter, Brea, CA, United States) with 1.3X Beads/DNA ratio for the “1st Bead Selection” and 1.6X Beads/DNA ratio for the “2nd Bead Selection” to obtain library size distributions in the range of 100–170bp. Library peak distribution was controlled and the average size was calculated using 1 μL on the Bioanalyzer High Sensitivity chip (Agilent Technologies, Santa Clara, CA, United States). The expected peak for miRNA is 147 bp. The 150 bp peak corresponds to piRNA. Molarity of the small RNA library was determined using KAPA Library Quantification Kit (Hoffman-La Roche, Basel, Switzerland) following the manufacturer’s recommendations. Sequencing was performed in 6-plex per sequencing lance on a HiSeq 4000. Bioinformatic analysis was performed with an in-house developed pipeline. Quality control was performed using fastqc (V0.11.3), the sequence reads were streamlined using Trimmomatic (V0.32), with a sequence quality threshold of Q30 and removing short reads (<15 bp). Reads were mapped using bowtie (V1.1.2) against mature and stem-loop miRNA databases (miRbase V21) as well as a proprietary in-house developed database, which takes natural variation of small RNA sequences into account. The expected peak size of mapped reads of a size peaking at 22–23 nucleotides was verified. Counts and TPM of analyzed miRNAs were recorded and filtered for very low expressed miRNAs (TPM < 10) in all samples. The filtered count file, with all miRNA with a TPM < 10 removed, was used for the differential analysis using DESeq2 (V1.6.3) (Love et al., 2014) and in-house developed R scripts were used to interpret and evaluate the results. R scripts were used for graphical representations such as heat maps and volcano plots.

### miRNA Target Prediction and Overrepresentation Test

Two different online databases were used for identifying target genes of differentially regulated miRNAs (*P* < 0.01). 2,458 miRNA targets were predicted using the TargetScan 7.2 software^3^. Additionally, 280 experimentally validated targets were identified using MiRTarBase 7.0 software^4^. For further analysis, a respective transcriptomic dataset (*P*_adj_ < 0.05) with 608 differentially expressed genes was included. Genes that were present in at least two of the three gene lists were analyzed for enriched biological processes using PANTHER-GO-Slim version 14.1. Fisher’s exact test was used to calculate p-values. The GO term analysis was corrected for a neutral background. Additionally, the Kyoto Encyclopedia of Genes and Genomes (KEGG) was used to include genes from cell cycle pathway (hsa0411) in the overlap analysis.

### Reverse Transcription and Semi-Quantitative Real-Time PCR

Gene expression of selected genes was validated using semi-quantitative real-time PCR (qRT-PCR) in a Step One Plus instrument (Thermo Fisher Scientific). 1,000 ng of extracted RNA of each condition were reverse transcribed using iScript^TM^ cDNA Synthesis Kit (Bio-Rad Laboratories, Inc., Hercules, CA, United States) according to the manufacturer’s protocol. For qRT-PCR analysis, SYBR Green Select qPCR Supermix (Thermo Fisher Scientific, Waltham, MA, United States), 5 ng complementary DNA from total RNA, 0.2 μM forward and reverse primers, and 0.1 μM 5-carboxy-X-rhodamine (passive references dye) were used. The PCR primer sequences are given in Supplementary Table 3. PCR was performed using the following protocol: 2 min at 50°C, 2 min at 95°C, and 40 cycles of 15 s at 95°C and 60 s at 60°C. Melting curves were recorded. Cycle threshold (C_*T*_) values were set within the exponential phase of the PCR. The correct size of the respective single amplicons was assured by agarose gel electrophoresis. Data was normalized to the four housekeeping genes, *ACTB*, *GAPDH*, *GPBP1*, and *RPL22*. Comparative normalized relative quantities (CNRQ) were used to calculate the relative expression levels using qBase Plus software (Biogazelle, Zwijnaarde, Belgium). Expression of miRNA hsa-miR-34c-5p was analyzed by miRCURY LNA Universal RT microRNA PCR system (Qiagen). RNA enriched fractions (50 ng) for each sample underwent reverse transcription according to the manufacturer’s protocol (Qiagen). Primers were purchased from Qiagen (hsa-miR-34c-5p, ID YP00205659; U6 snRNA, ID YP00203907) and *RNU6* was used as a reference gene for normalization. Results were evaluated by quantitative real-time PCR (StepOne Plus, Applied Biosystems) and analyzed using the 2-ΔΔCT method as described in the manufacturer’s manual (Applied Biosystems).

### Cytotoxicity Assay

On day 6 or 8 of differentiation, 30 μL of medium of each well were transferred to a 96 well plate and 70 μL of 80 mM Tris/HCl/200 mM NaCl (pH 7.2) buffer containing 10 mM NADH and 100 mM pyruvate (Sigma-Aldrich) was added. In the assay, lactate dehydrogenase (LDH) converts pyruvate to lactate by consuming NADH. NADH metabolization is proportional to LDH in the medium and its absorption at 340 nm was monitored with a reference measurement at 420 nm (absorption minimum for NADH) by a spectrophotometer (ClarioStar, BMG labtech, Ortenburg, Germany).

### Cell Viability Assay

Calcein acetoxy methylester (-AM) is a cell-permeable dye. It was used to determine cell viability in 6 and 8 days differentiated cells. In viable cells, the non-fluorescent calcein-AM is converted to green-fluorescent calcein by intracellular esterases. The dye was added into the medium at a 1:1,000 dilution. The reaction plate was incubated for 30 min at 37°C. The medium was replaced with 50 μL of PBS and the fluorescence intensity measured at λ = 577/619 nm by a spectrophotometer (ClarioStar, BMG Labtech, Ortenburg, Germany) using well scan mode with a 9 × 9 matrix.

### Small Interfering RNA Treatment With siPOOLs

Cells were treated with OptiMem medium containing lipofectamine RNAiMax (Thermo Fisher Scientific) and siPOOLs (SiTools, Martinsried, Germany) comprising 20 individual small interfering RNAs (siRNAs) harboring different seeding sequences against a respective mRNA species, thereby minimizing off-target effects. For the treatment, either 96-well (100 μL medium) or 6-well (2 mL medium) plates were used. For each condition, different concentrations of siRNAs were tested in αSyn-transduced LUHMES neurons. Expression levels of each target were analyzed with qRT-PCR to determine a working concentration which leads to comparable silencing efficiency for cell cycle gene cyclin D1 (*CCND1*) (50 nM), *CCND2* (5 nM) or both in combination.

### Statistical Analysis

GraphPad Software Prism 7 (GraphPad Software, La Jolla, CA, United States) was used for statistical analysis. Each experiment includes at least three independent repeats. Data are presented as mean ± standard error of the mean (SEM). Data were generally compared by ordinary two−way ANOVA with Bonferroni’s or Sidak’s *post hoc* test, unless otherwise indicated. If statistically significant, pairwise comparisons were evaluated by a two-tailed unpaired *t*-test.

## Results

### Study Overview

**FIGURE 1:**
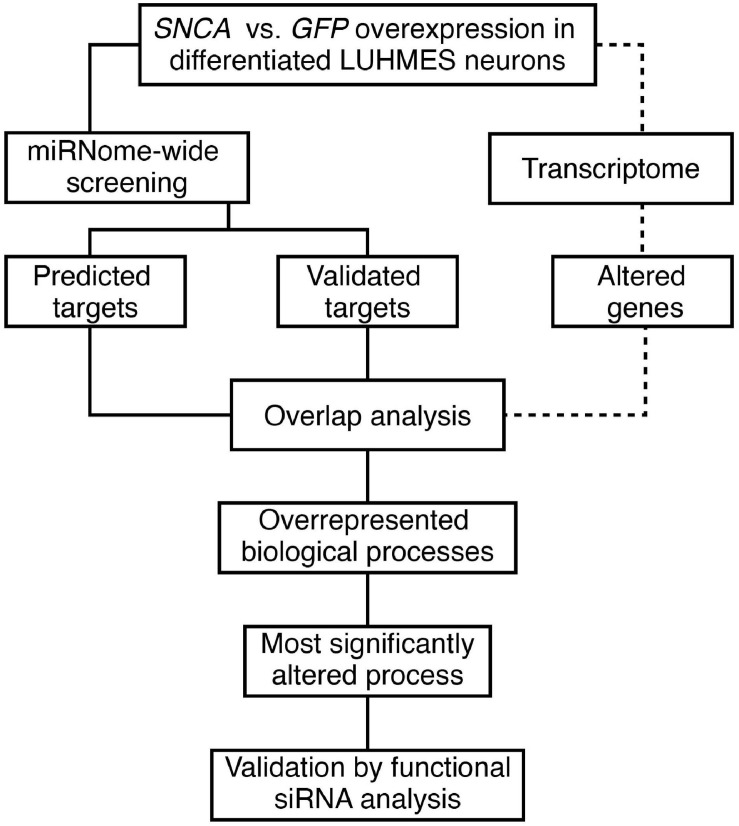
Overview of the study workflow. An *in vitro SNCA* overexpression model of αSyn-related pathology compared to a GFP-control model served as basis for the identification and analysis of miRNA target genes.

We studied the effect of early phases of αSyn pathology on the miRNome in a human neuronal cell model using a hypothesis-free approach. Figure 1 presents an overview of the analytical process. A miRNome-wide screen was performed in LUHMES neurons overexpressing either αSyn or GFP 4 days post transduction. The resulting differentially expressed miRNAs with significance (*P* < 0.01) were analyzed further using two public databases, TargetScan 7.2 and MiRTarBase 7.0. Predicted and experimentally validated gene targets of the miRNA hits were compared by an overlap analysis with the respective transcriptome data set (BeadChip). Genes present in at least two overlapping entities were used for enrichment analysis. The most significantly enriched biological process was analyzed by validation and subsequent siRNA-mediated functional analysis in the *SNCA* overexpression model.

### *In vitro SNCA* Overexpression Model

**FIGURE 2:**
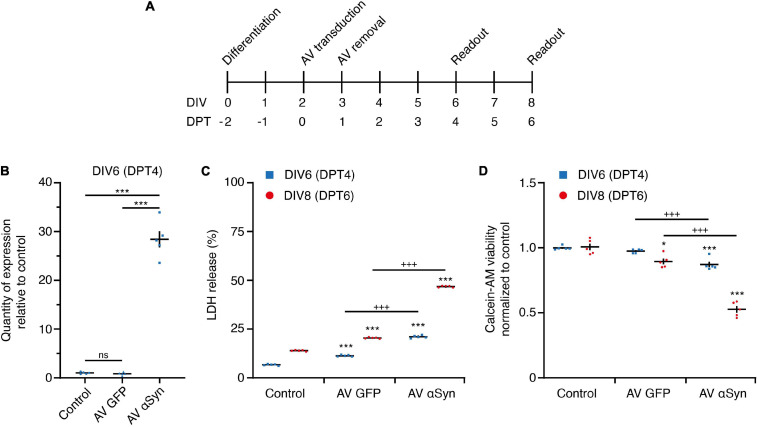
*In vitro SNCA* overexpression model. **(A)** *In vitro* treatment scheme. LUHMES neurons were transduced with adenoviral (AV) vectors of SNCA or GFP (control virus). DIV, days *in vitro*; DPT, days post transduction. **(B)** Expression of *SNCA* mRNA in LUHMES neurons after 4 days of transduction with GFP AV or SNCA AV vectors. **(C)** Cytotoxicity measured by release of lactate dehydrogenase (LDH) at indicated read-out times in untreated cells (control) and cells transduced with AV GFP (virus control) or AV SNCA. LDH release is expressed as percentage of lysed cells (positive control). **(D)** Cell viability measured by intracellular hydrolysis of calcein acetoxy methyl (-AM) ester at indicated read-out times in untreated cells (control) and cells transduced with AV GFP (virus control) or AV SNCA. Data of AV GFP and AV SNCA were normalized to the respective readout control, set as one. Results are presented as mean ± SEM from at least three biological repeats. Statistical analysis in panel **(B)** was Students *t*-test and in panels **(C,D)** ordinary two-way ANOVA with Bonferroni’s multiple comparison test. ^+++^*P* < 0.001; *P < 0.05, ***P < 0.001 vs. respective control.

In order to validate the usability of our *in vitro* model, we determined different parameters. Cells were transduced with αSyn adenoviruses (AV) after 2 days *in vitro* (DIV) and we determined toxicity, viability and *SNCA* expression levels at DIV6 and DIV8 days (Figure 2A). A strong *SNCA* overexpression [log2 fold change (FC) = 4.83; *P* < 0.001] was detected by qRT-PCR 4 days post transduction (DPT; Figure 2B). The overexpression-induced toxicity, measured by extracellular LDH activity, significantly increased over time (Figure 2C) and reached 21.1 ± 0.31% on DIV6 and 46.8 ± 0.20% on DIV8. The cell viability, measured by the amount of calcein fluorescence, was significantly decreased in *SNCA*-overexpressing neurons to 88 ± 2% on DIV6 and to 54 ± 3% on DIV8 (Figure 2D) in comparison to the respective controls. A disruption of the cell differentiation process or the presence of non-neuronal cell populations by SNCA overexpression could be excluded (see Supplementary Figures 1,2).

In summary, these results confirm the successful adenoviral *SNCA* transduction, emerging toxicity and reduced viability as a measure of αSyn-mediated neurodegeneration at DIV6 (= DPT4). Thus, we used this time point to study the miRNome in greater detail.

### miRNome-Wide Screen and Overlap Analysis With Transcriptome Data

**FIGURE 3:**
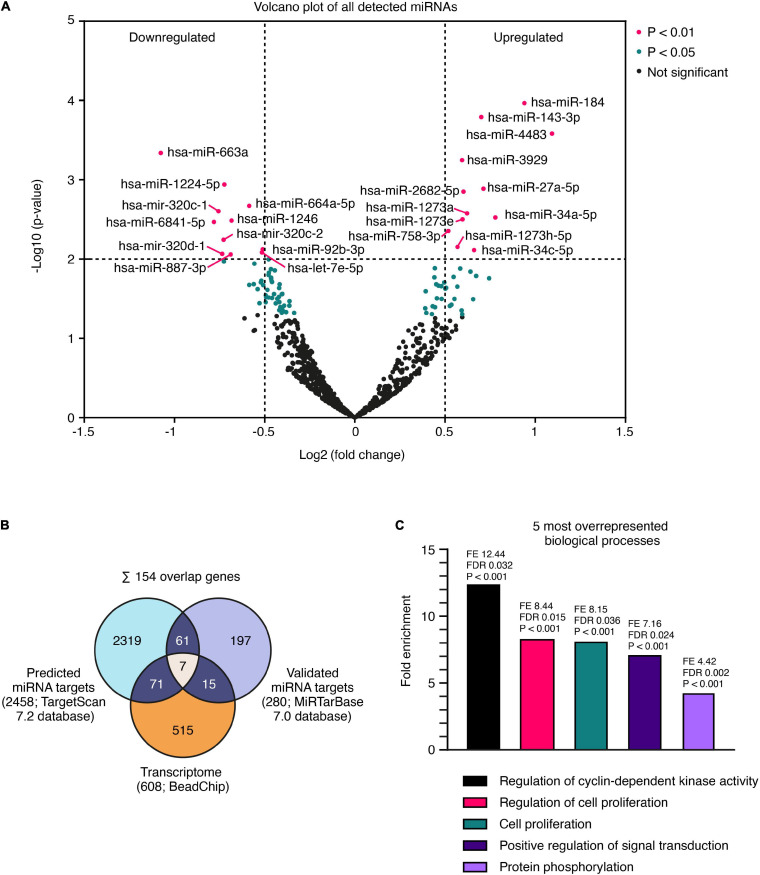
miRNome-wide screen and overlap analysis with transcriptome data. **(A)** Volcano plot shows 796 detected miRNAs in *SNCA* vs. *GFP* overexpressing LUHMES neurons 4 days after adenoviral transduction. 55 miRNAs with *P* < 0.05 and 23 with *P* < 0.01 were differentially expressed (non-adjusted p-value). **(B)** Identification of 154 overlap genes of predicted miRNA target genes (TargetScan 7.2 database) and experimentally validated target genes (MiRTarBase 7.0 database) with miRNAs of *P* < 0.01, and significantly regulated mRNAs (*P*_adj_ < 0.05; Benjamini–Hochberg FDR) from transcriptome analysis (BeadChip). **(C)** Overrepresented biological processes identified by PANTHER GO-Slim pathway analysis among 154 overlap genes.

Aberrant miRNA expression of incipient αSyn-mediated neurodegeneration was studied by a hypothesis-free comprehensive miRNome-wide approach using next-generation sequencing at DPT 4. Among 798 detected miRNAs, 55 were found differentially expressed at *P* < 0.05 including 23 significantly (*P* < 0.01; non-adjusted) altered compared to GFP controls. Among the 23 miRNAs with significance (*P* < 0.01), 11 miRNAs were downregulated and 12 miRNAs upregulated (Figure 3A; see Supplementary Table 1 for more details). Out of these 23 miRNAs, 13 were found without prior evidence for a relation to synucleinopathies while 10 miRNAs had already been linked to PD (Supplementary Table 1). A comprehensive *in silico* analysis of these 23 altered miRNAs was performed using TargetScan 7.2 and MirTarBase 7.0. A total of 2,458 predicted and 280 experimentally validated messenger RNAs have been identified as probable target genes (Figure 3B). According to MirTarBase 7.0, hsa-miR-34a-5p and hsa-miR-34c-5p directly target *SNCA* mRNA. Predicted and experimentally validated gene targets of 23 significantly altered miRNAs were compared by an overlap analysis with a respective transcriptome data set from our cell model that included 608 genes that were significantly differentially expressed in comparison between aSyn overexpressing and GFP expressing cells (*P*_adj_ < 0.05). We found 154 genes present in at least two of the three gene lists. Those 154 genes were further tested for enriched biological processes with PANTHER GO-Slim (Figure 3C; see Supplementary Table 2 for more details). We discovered the highest enrichment for the G0/G1 cell cycle regulation of cyclin-dependent protein serine/threonine kinase activity (12.44 fold; FDR 3.17E-02) followed by regulation of cell proliferation (8.36 fold; FDR 1.46E-02), cell proliferation (8.15 fold; 3.58E-02), positive regulation of signal transduction (7.16 fold; 2.39E-02), and protein phosphorylation (4.42 fold; 2.49E-03). Within the highest enriched pathway, we discovered an overrepresentation of target genes *CCND1*, *CCND2*, *CCNE2*, and *CDKN1C* involved in the G0/G1 cell cycle superpathway.

### Expression of Cell Cycle Genes Involved in the G0/G1 Superpathway

**FIGURE 4:**
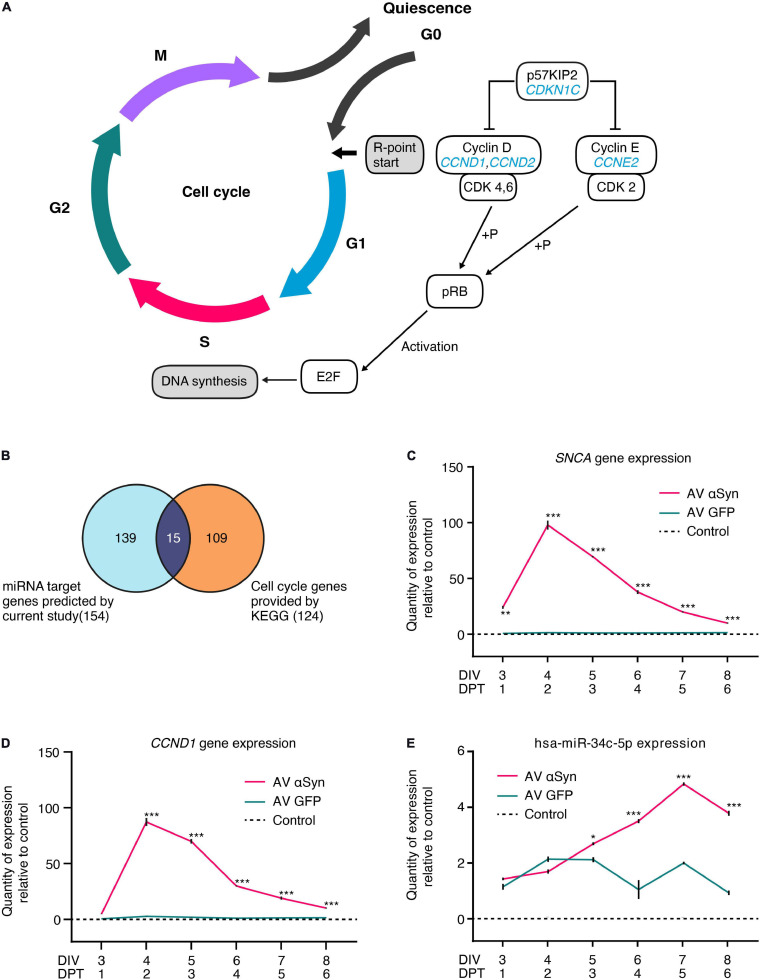
Cell cycle as most significantly altered process. **(A)** Simplified illustration of the mitotic cell cycle phases. Encoded proteins of the genes *CCND1*, *CCND2*, *CCNE2*, and *CDKN1C* (labeled in blue) from the most significantly enriched process after PANTHER GO-Slim pathway analysis are key players for the transition of the cell from quiescence to initiation of G1 cell cycle phase. **(B)** Overlap of 154 target genes predicted by current study and 124 KEGG (Kyoto Encyclopedia of Genes and Genomes) cell cycle genes identified 15 *SNCA* overexpression-affected cell cycle genes. Gene expression of *SNCA* **(C)**, *CCND1* **(D)**, and *hsa-miR-34c-5p* **(E)** measured over time by qRT-PCR in LUHMES neurons transduced with adenoviral (AV) vectors of SNCA or GFP (control virus) or non-transduced (control). Values are related to non-transduced control at the corresponding times set as one. Values in panels **(C–E)** are mean ± SEM from at least three biological repeats. Statistical analysis was performed by a two-way ANOVA with Sidak’s correction for multiple comparison testing and, if significant, pairwise comparisons were evaluated by an unpaired *t*-test. *P < 0.05, **P < 0.01, ***P < 0.001 vs. GFP virus control in panels **(C–E)**; DIV, days *in vitro*; DPT, days post transduction.

According to the KEGG (Kyoto Encyclopedia of Genes and Genomes) database, *CCND1*, *CCND2*, *CCNE2*, and *CDKN1C* are required for the G0/G1 cell cycle transition (Figure 4A). We compared 124 KEGG-annotated cell cycle genes with 154 miRNA target genes of our dataset (Figure 4B). Out of 15 overlapping genes, 10 genes were annotated for G0/G1 cell cycle phases. We confirmed significant differential expression levels of six genes in *SNCA*-overexpressing human post-mitotic midbrain-derived neurons that are involved in the G0/G1 cell cycle pathway (Table 1). Five genes were upregulated: *CCND1*, *CCND2, CDK4, E2F3, MYC*; and one gene was downregulated: *CDKN1C*. According to MirTarBase, several cell cycle genes are also targeted by hsa-miR-34a-5p and hsa-miR-34c-5p, which have been shown to directly target *SNCA*.

**TABLE 1:** Gene expression of 10 G0/G1 cell cycle-related genes obtained from the transcriptome analysis (BeadChip) and validated by qRT-PCR analysis.

**Gene**	**Transcriptome data**		**qRT-PCR data**	
	**Log2 FC**	**P_adj_**	**Log2 FC**	***P***
CCND1*	0.86	0.035	5.09	0.0018
CCND2*	1.98	0.006	2.17	0.0007
*CCNE2*	n.d.	–	0.08	n.s.
CDK4*	0.51	0.043	1.12	0.0082
*CDK6*	n.d.	–	0.31	n.s.
*CDKN1A, p21*	1.35	0.022	0.26	n.s.
*CDKN1C, p57*	n.d.	–	−0.36	0.0004
E2F3*	0.59	0.042	0.87	0.0121
*E2F5*	n.d.	–	0.15	n.s.
*MYC*	0.06	–	0.98	0.012

*significantly altered in both data sets; n.d., not detected; n.s., not significant.

In order to observe effects of αSyn overexpression on the most upregulated target gene *CCND1* (log_2_FC = 5.09, *P* < 0.005), we monitored the expression levels of *SNCA* and *CCND1* every 24 h, starting from DIV3 until DIV8 (Figures 4C,D and Supplementary Figures 3A,B). Expectedly, relative quantity (RQ) of *SNCA* expression increased significantly stronger between DIV3 (24.1 RQ ± 1.6 SEM; *P* = 0.001) and DIV4 (97.9 RQ ± 4.1 SEM; *P* < 0.001) and decreased thereafter until DIV8 (10.0 RQ ± 0.5 SEM; *P* < 0.001) in *SNCA* AV-transduced cells compared to the *GFP* AV-transduced controls (Figure 4C and Supplementary Figure 3A). We found that *CCND1* expression showed a very similar expression curve to *SNCA* with a most significant increase between DIV3 (4.9 RQ ± 0.3 SEM; *P* < 0.001) and DIV4 (87.4 RQ ± 3.5 SEM; *P* < 0.001) and a decrease until DIV8 (10.1 RQ ± 0.5 SEM; *P* < 0.001) (Figure 4D and Supplementary Figure 3B).

Within the same time frame, we additionally examined the expression of *SNCA*-regulating miRNA hsa-miR-34c-5p which was significantly upregulated (*P* < 0.01) in the miRNome-wide screen on DIV6 (Figure 4E). We found that hsa-miR-34c-5p expression significantly increased between DIV5 (2.7 RQ ± 0.03 SEM; *P* = 0.0164) and DIV7 (4.84 RQ ± 0.06 SEM; *P* < 0.001) and decreased on DIV8 (3.8 RQ ± 0.9 SEM; *P* < 0.001) in AV SNCA samples compared to AV GFP control. While *SNCA* expression steadily decreased after peaking on DIV4 (Figure 4C), hsa-miR-34c-5p expression was significantly upregulated in AV αSyn-treated cells. In summary, the expression analysis showed that altered cell cycle gene expression might be correlated with *SNCA* overexpression. We also validated the miRNA screening result for *SNCA*-regulating miRNA hsa-miR-34c-5p on DIV6 and observed that 1 day after *SNCA* expression peaked, miRNA expression was significantly increased.

### Functional Analysis of Cyclin D1 and D2 Gene Silencing

**FIGURE 5:**
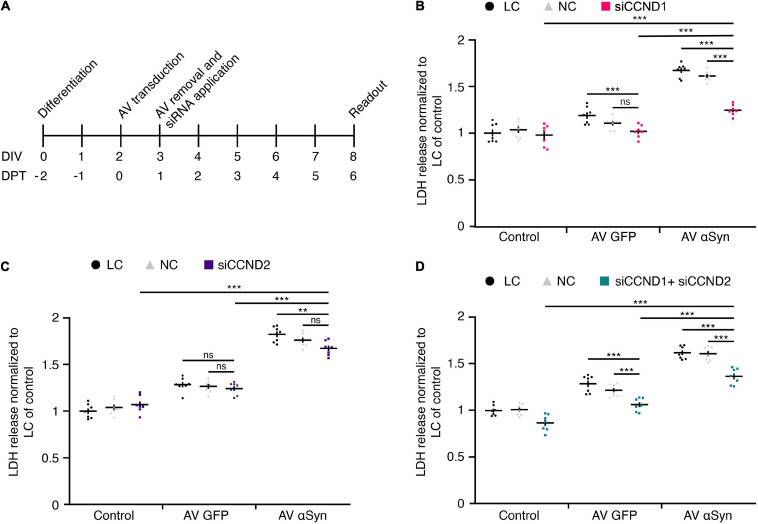
Functional siRNA analysis. **(A)** Experimental treatment scheme. LUHMES neurons were transduced with adenoviral (AV) vectors of SNCA or GFP (control virus) and treated with or without siRNAs against *CCND1* and/or *CCND2*. LDH release as measure for cytotoxicity was determined in control 6 days after transduction. DIV, days *in vitro*; DPT, days post transduction. **(B–D)** Quantification of cytotoxicity upon treatment with siCCND1 **(B)**, siCCND2 **(C)**, and siCCND1 + siCCND2 **(D)**. Experiments in panels **(B–D)** included lipofectamine controls (LC), non-coding siRNA controls (NC) in parallel to the coding siRNA treatment. Data was normalized to LC in control cells. Results are presented as mean ± SEM from seven biological repeats. Statistical analysis in panels **(B–D)** was performed by a two-way ANOVA with Bonferroni’s correction for multiple testing and, if significant, pairwise comparisons were evaluated by an unpaired *t*-test. **P < 0.01, ***P < 0.001; ns, not significant.

To test the relevance of upregulated cell cycle genes in αSyn-mediated toxicity, we proceeded with a functional analysis of *CCND1* and *CCND2* using a siRNA approach. As these particular cell cycle genes were found to be most upregulated upon *SNCA* overexpression on DIV6 (= DPT4) in differentiated LUHMES neurons, we silenced each gene separately and in combination on DIV3 (Figure 5A). In order to ensure comparable mRNA levels between the targets, silencing efficiency of siRNA against *CCND1* and *CCND2* separately and in combination was assessed using qRT-PCR (Supplementary Figures 4A–C).

On DIV8 (= DPT6), when the viability of *SNCA*-overexpressing neurons was reduced to ∼50% (Figures 2C,D), cytotoxicity was analyzed by measuring LDH release into the medium.

Treatment with 50 nM siRNA directed against *CCND1* (1.24 ± 0.02) significantly reduced αSyn-induced toxicity compared to lipofectamine control (LC) (1.66 ± 0.03; *P* < 0.0001) and non-coding (NC) control siRNA (1.60 ± 0.02; *P* < 0.0001) (Figure 5B). The *CCND2* (1.68 ± 0.03) siRNA treatment at 5 nM did not significantly alter αSyn-induced toxicity in NC (1.76 ± 0.03; n.s.), only in LC (1.82 ± 0.03; *P* < 0.0087) control (Figure 5C). However, the combined application of targeting siRNAs against *CCND1* and *CCND2* (1.38 ± 0.03) significantly decreased toxicity levels under LC (1.62 ± 0.03; *P* < 0.0001) and NC (1.61 ± 0.03; *P* < 0.0001) conditions in *SNCA* virus-transduced cells (Figure 5D). In summary, siRNA silencing of the top-most upregulated cell cycle gene *CCND1* identified through a comprehensive miRNA screen led to considerable attenuation of LDH release in *SNCA* overexpressing post-mitotic neurons by 38.7%.

## Discussion

In the present study, we performed a comprehensive miRNome-wide screen in human post-mitotic midbrain derived LUHMES neurons with moderate αSyn-induced cytotoxicity levels. We discovered a significant differential expression of 23 miRNAs (*P* < 0.01; non-adjusted) with 12 miRNAs- so far without–and 11 miRNAs with known relation in the scientific literature to models for synucleinopathies or PD. By including comprehensive *in silico* target gene mining and subsequent bioinformatics analysis of target genes, we observed an enrichment of the cell cycle superpathway and confirmed significant differential expression levels of six cell cycle genes in *SNCA*-overexpressing post-mitotic neurons. By looking into the daily evolvement of the *SNCA* transcriptional upregulation between DIV3 and DIV8, we noted a very similar expression curve of *CCND1* and *SNCA*, whereas the expression of *CCND1* was not altered in *GFP* expressing control cells. We also validated the upregulation of *SNCA*-regulating miRNA hsa-miR-34c-5p in a time-delayed manner after *SNCA* expression peaked. Silencing of the two most upregulated genes *CCND1* and *CCND2*, involved in the G0/G1 cell cycle initiation, revealed that siRNA against *CCND1* alone and in combination with *CCND2* markedly reduced cytotoxicity levels. Taken together, early-stage intracellular accumulation of αSyn in human mesencephalic post-mitotic neurons (Chakroun et al., 2020) is accompanied by an altered expression of miRNAs, leading to an enrichment of G0/G1 cell cycle genes. Among them, *CCND1* seems to be functionally involved in αSyn-mediated cell death, as shown by siRNA-mediated intervention.

In our robust model for αSyn-mediated pathology, we found the levels of 23 miRNAs to be significantly altered. Out of these 23 miRNAs, 12 have not been implicated in the pathogenesis of synucleinopathies and related models so far. Interestingly, 11 miRNAs (hsa-miR-27a-5p, hsa-miR-34a-5p, hsa-miR-34c-5p, hsa-let-7e-5p, hsa-miR-184, hsa-mir-320c-1, hsa-mir-320c-2, hsa-miR-320d-1, hsa-miR-1224-5p; hsa-miR-92b-3p; and hsa-miR-887-3p) have already been reported to undergo dysregulation in PD patients or been linked to either target *SNCA* or pathophysiological mechanisms of PD (Minones-Moyano et al., 2011; Sibley et al., 2012; Soreq et al., 2013; Briggs et al., 2015; Kabaria et al., 2015; Prajapati et al., 2015; Hoss et al., 2016; Shamsuzzama et al., 2017) (see Supplementary Table 1). These results substantiate the validity of our model. Our data is largely confirmed by further studies performed in other PD models. For instance, in dopaminergic neurons of PD patients, an upregulation of hsa-miR-27a and hsa-miR-184 was reported in accordance with our *SNCA* overexpression PD model (Briggs et al., 2015). In leukocytes of PD patients, hsa-mir-320c and hsa-miR-92b-3p were found to be downregulated similarly to the results of the present screen (Soreq et al., 2013). Additionally, we found a downregulation of hsa-miR-1224-5p and hsa-miR-887-3p, which have been reported to be downregulated in the prefrontal cortex of PD patients (Sibley et al., 2012; Hoss et al., 2016). However, some of our findings are not in line with previously reported data. We report an upregulation of both, hsa-miR-34a-5p and hsa-miR-34c-5p, which have been shown to directly modulate *SNCA* expression in another cell model of PD (Kabaria et al., 2015). By monitoring the expression of *SNCA* and hsa-miR-34c-5p between DIV3 until DIV8, we not only validated the upregulation found in the miRNA screen but also showed that hsa-miRNA-34c-5p was significantly upregulated after maximal SNCA expression was reached on DIV4. While *SNCA* expression steadily decreased thereafter, hsa-miRNA-34c-5p stayed upregulated until DIV8. Since hsa-miR-34c can inhibit *SNCA* expression by targeting its 3′-UTR (Kabaria et al., 2015), the upregulation of hsa-miR-34c in our study might be a counter-regulatory measure of the cell to lower the increased αSyn levels. In postmortem brain tissue of PD patients at a late disease stage hsa-miR-34c was shown to be downregulated (Minones-Moyano et al., 2011). This controversial finding could be explained by the fact that we used a cell model of early αSyn overload. Therefore, one could speculate that either miR-34c expression levels might change depending on disease stage and/or are influenced by non-neuronal cell populations, as unsorted postmortem brain tissues (including glial cells) was used in the tissue analysis. Furthermore, hsa-let-7 miRNA has been shown to be upregulated upon oxidative stress in other cell models of PD (Prajapati et al., 2015; Shamsuzzama et al., 2017), while we observed a downregulation in our model. This rather opposite finding could be attributed to the fact that the complex miRNA expression profile varies depending on the induced stress, duration and species in different PD models such as human SHSY-5Y cells and dopaminergic neurons of *C. elegans*. The levels of other PD-associated miRNA, such as miR-7, miR-153 or miR-133b, were not significantly differentially expressed in our *SNCA* overexpression model. Since we were modeling an early-stage αSyn-mediated pathology in dopaminergic neurons, those miRNAs could be either involved at other stages or in other cell types relevant for the disease or might be model specific.

A single miRNA might affect several different mRNAs encoding distinct target genes with a tissue specific expression profile (Halushka et al., 2018). Hence, investigation and interpretation of miRNA-mRNA interactions remains very challenging. In our *SNCA* overexpression model, we identified cell cycle and proliferation processes to be overrepresented. αSyn is a key protein in PD pathogenesis, but its physiological function is still not fully understood. In addition to its presence at the synapse, αSyn has been confirmed to be localized in the nucleus (Goncalves and Outeiro, 2013). Due to its DNA-binding properties, a regulating role in transcription has been suggested (Martins et al., 2011) which could result in the miRNA and gene expression alterations observed in our model. Furthermore, in accordance with our findings, an emerging body of evidence has linked PD and αSyn to cell cycle processes (Lee et al., 2003; Höglinger et al., 2007; Ma et al., 2014; Paiva et al., 2017; Sampaio-Marques et al., 2019). An increased number of proteins associated with DNA synthesis and cell cycle re-entry was reported in dopaminergic neurons of postmortem PD brain tissue (Höglinger et al., 2007). Similar observations were made in cellular and animal models of PD (Lee et al., 2003; Paiva et al., 2017). The atypical re-entry of post-mitotic dopaminergic neurons into the cell cycle is believed to cause apoptosis (Sampaio-Marques et al., 2019), as well as nuclear accumulation of αSyn in PC12 cells (Ma et al., 2014). Both of these effects were shown to promote neurotoxicity through cell cycle activation (Ma et al., 2014). Therefore, our finding of cell cycle gene alterations and increased cytotoxicity upon αSyn overload are in accordance with reports from PD patients and other PD models. Yet, the exact mechanism leading to a deleterious cell cycle activation remains unknown.

The control of cell proliferation by distinct miRNAs has predominantly been described in cancer (Lin and Gregory, 2015). Cell cycle-related genes are regulated by several miRNAs, which were also present in our post-mitotic neuronal cell model. Out of the 23 significantly altered miRNAs detected in this study, eight miRNAs (hsa-let-7e-5p, hsa-miR-184, hsa-miR-34a-5p, hsa-miR-34c-5p, hsa-miR-92b-3p, hsa-miR-1246, hsa-miR-143-3p, and hsa-miR-663a) had already been experimentally validated to regulate cell cycle-related genes (Hermeking, 2010; Mitra et al., 2011; Wu et al., 2013; Zhang et al., 2013; Zhen et al., 2013; Chai et al., 2016; Cho et al., 2016). Interestingly, five of those (hsa-let-7e-5p, hsa-miR-184, hsa-miR-34a-5p, hsa-miR-34c-5p, and hsa-miR-92b-3p) had already been implicated in synucleinopathies (Soreq et al., 2013; Briggs et al., 2015; Kabaria et al., 2015; Prajapati et al., 2015; Shamsuzzama et al., 2017). The rest of the miRNAs listed in Figure 3A had, to our knowledge, not yet been reported in the context of synucleinopathies. For example, in different cancer types the let-7 miRNA family alone modulates the abundance of *CCND1, CDK4* as well as *MYC* (Mitra et al., 2011; Fairchild et al., 2019). *MYC* was also reported to regulate the expression of a number of miRNAs, e.g., hsa-let-7 and hsa-miR-34a (Bueno and Malumbres, 2011). The complexity of this miRNA-mRNA network presents a challenge to the interpretation of our study results. Thus, one could speculate that the differential miRNA expression pattern we report might not only occur in response to the αSyn-mediated cell cycle alterations, but also contribute to these alterations. However, as both hsa-miR-34a-5p and hsa-miR-34c-5p not only target *SNCA* (Kabaria et al., 2015) but also *CCND1* (Sun et al., 2008; Achari et al., 2014), the upregulation of both miRNAs could be a response to repress the expression of both. The decreasing expression levels of *SNCA* and *CCND1* after the upregulation of their regulating miRNA hsa-miR-34c-5p between DIV5 until DIV8, point toward a counter-regulatory role of this specific miRNA in our model. Furthermore, due to the diverse roles of miRNAs in cellular processes, biological mechanisms apart from cell cycle activation such as the positive regulation of signal transduction and/or protein phosphorylation must certainly be considered. Indeed, both processes were also found to be markedly enriched in our miRNome-wide screen. As this study did not investigate the immediate effect of the differentially regulated miRNAs on CCND1 and SNCA expression, additional functional studies of miRNAs, e.g., hsa-miR-34a-5p, hsa-miR-34c-5p, and hsa-let-7e-5p, are required to unravel their role in αSyn-mediated cell cycle alterations and cytotoxicity.

However, as our results were obtained in post-mitotic neurons, additional functions such as stress-related cell cycle induction should be considered. Upon cellular stress, the activation of the transcription factor p53 was shown to change the expression of several genes encoding miRNAs (Olejniczak et al., 2018). For instance, miRNAs hsa-miR-34a-5p and hsa-miR-34c-5p, which were upregulated in our model, were described to be regulated by p53 in breast cancer cells (Javeri et al., 2013). It is conceivable that intracellular accumulation of αSyn and subsequent aggregation might lead to cellular stress and a p53-mediated change in miRNA levels. Further studies are needed to determine possible functional relations and sequence of events of αSyn, p53 and miRNAs.

Cell cycle activation depends on the delicate balance of mitogenic factors. Growth factors stimulate the expression of crucial G1 phase genes, such as *CCND1, CCND2, CDK4*, which were upregulated in our model, whereas the cell cycle inhibitor p57/Kip2 (*CDKN1C*) was found to be downregulated. During the G1 phase, cyclin D protein binds to cyclin-dependent kinases 4 or 6 (CDK4/6) and, through the neutralization of retinoblastoma protein (Rb), activates E2F transcription factor which in turn initiates cell cycle progression by regulating the transcription of S-phase genes (Figure 4; Aktas et al., 1997; Choi and Anders, 2014). In *SNCA*-overexpressing neurons, we found an elevated expression of *CDK4* as well as one gene of the E2F family (*E2F3*; Table 1). The passage of different cell cycle checkpoints usually prevents uncontrolled activation of the cell cycle machinery. However, a dysfunction could be compensated by the induction of apoptosis (Pardee, 1974). In the developing brain, the balance between cell proliferation and programmed cell death is crucial for establishing a functional neural network. Both processes are highly conserved and share common mediators (Pucci et al., 2000). During maturation, cells no longer undergo mitosis, become post-mitotic and terminally differentiated. Therefore, our finding that *SNCA* overexpression in post-mitotic neurons leads to altered expression of genes responsible for cell cycle activation was rather unexpected. As *CCND1* expression in *SNCA* overexpressing human neurons over time was similar to *SNCA* levels and successful silencing of *CCND1* reduced neuronal cell death, a correlation between aberrant cell cycle entry and αSyn-induced neurodegeneration might be well indicated in our model. The downstream effects of aberrant cell cycle activation in post-mitotic neurons resulting in cell death have already been established in multiple studies (Freeman et al., 1994; Padmanabhan et al., 1999; Ino and Chiba, 2001; Jordan-Sciutto et al., 2003; Sumrejkanchanakij et al., 2003; Herrup and Yang, 2007; Höglinger et al., 2007; Pinho et al., 2019; Sampaio-Marques et al., 2019). Our findings suggest that miRNA dysregulation upon *SNCA* overexpression might be a very upstream activation mechanism for cell cycle re-activation in post-mitotic neurons. In order to conclusively establish the functional role of the reported altered miRNAs in the activation or counter-regulation of the cell cycle machinery, further studies, such as targeted knockdown of specific miRNAs, will be required.

Apart from its cell cycle regulatory functions, several other roles of cyclin D should be considered. Cyclin D1 has been shown to inhibit the mitochondrial metabolism (Sakamaki et al., 2006). Mitochondrial dysfunction is currently proposed as a central factor in PD pathogenesis (Franco-Iborra et al., 2016). Additionally, cyclin D was shown to play a critical role for chromatin remodeling (Hulit et al., 2004), another epigenetic mechanism that has been associated with PD pathophysiology (Labbe et al., 2016). Higher levels of histone acetylation have been found in PD patients (Park et al., 2016) and inhibition of histone acetylases was shown to protect against αSyn-induced toxicity (Kontopoulos et al., 2006). The neuroprotective effect of *CCND1* silencing that we report might be mediated by its role in chromatin remodeling. As cyclin D governs diverse roles in the cytoplasm and cell nuclei, further investigations are needed to determine the exact mechanisms underlying the interplay between cyclin D and αSyn-mediated pathology.

In conclusion, a miRNome-wide screen at an early time point of *SNCA* overexpression in post-mitotic human neurons resulted in 23 differentially regulated miRNAs, including 13 novel miRNAs in the context of PD. Target gene analysis of the respective miRNAs revealed an enrichment of cell cycle G0/G1 activation, which we confirmed using qPCR analysis. Silencing of *CCND1* under *SNCA* overexpression conditions proved to be protective and significantly reduced neurotoxicity. Altogether, our findings reveal that targeting miRNA regulation of the cell cycle pathway in post-mitotic neurons offer a viable therapeutic approach for PD and eventually other synucleinopathies. Notwithstanding, further research is necessary to determine the exact pathways and mechanisms involved in aberrant cell cycle re-entry, and to confirm *CCND1* and its regulating miRNAs as novel drug targets to modulate pathophysiology in PD.

## Data Availability Statement

Publicly available datasets were analyzed in this study. This data can be found here: www.targetscan.org and http://miRTarBase.mbc.nctu.edu.tw/. Other data used to support the findings of this study are included in the Supplementary Material. If any other data are needed, please contact the corresponding author.

## Author Contributions

EF, SS, and GH conducted the project design. EF, YS, and JT carried out experiments. EF, YS, JT, SS, N-PN, TC, and VE analyzed the data. MH and MK provided the transcriptomic data. EF, SS, and TR drafted the manuscript and conceptualized the figures. All authors corrected and approved the final manuscript.

## Conflict of Interest

The authors declare that the research was conducted in the absence of any commercial or financial relationships that could be construed as a potential conflict of interest.

## Footnotes

1 https://www.knime.com/

2 https://www.bioconductor.org/

3 http://www.targetscan.org

4 http://mirtarbase.mbc.nctu.edu.tw
