# Supplementary material for: Comprehensive miRNome-Wide Profiling in a Neuronal Cell Model of Synucleinopathy Implies Involvement of Cell Cycle Genes
Source: Front Cell Dev Biol. 2021 Mar 4;9:561086. doi: 10.3389/fcell.2021.561086 (PMC7969723; doi:10.3389/fcell.2021.561086)
Supplement: Supplementary file 1 [file Data_Sheet_1.docx]

Supplementary Material

# Supplementary Figures


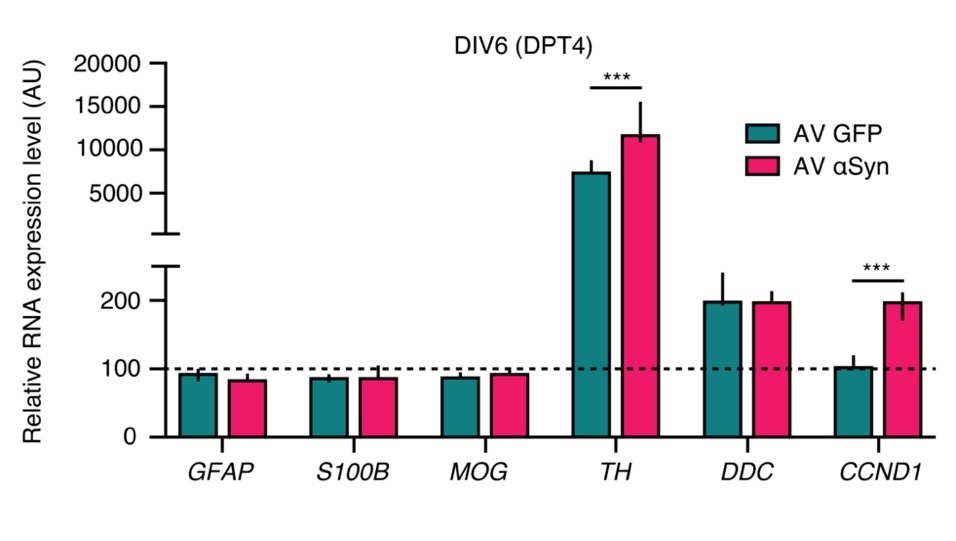


**Supplementary Figure 1.** mRNA expression analysis at 6 days *in vitro* (DIV6), 4 days post transduction (DPT4) of LUHMES neurons transduced with adenoviral (AV) vectors, either expressing alpha-synuclein (AV αSyn) or GFP (AV GFP) as an overexpression control. The dashed line indicates the lower limit of the detection threshold. In fully differentiated LUHMES cells, specific mRNA expression of glial markers such as *GFAP*, *S100B*, *MOG* could not be observed (levels below the lower limit of the detection threshold). Tyrosine hydroxylase (*TH*) mRNA levels were higher in αSyn expressing cells, whereas expression levels of dopa-decarboxylase (*DDC*) mRNA were unaltered, suggesting that αSyn overexpression did not negatively influence dopaminergic differentiation. Expectedly, also the chip analysis showed an increase of *CCND1* mRNA in αSyn-overexpressing cells compared to cells expressing GFP as control. Relative expression levels are shown as arbitrary units (AU) (median with upper and lower quartiles) from 3 biological repeats. Statistical analysis was 1-way analysis of variance followed by *post hoc* Tukey test. ***P < 0.001 AV GFP vs. AV αSyn.


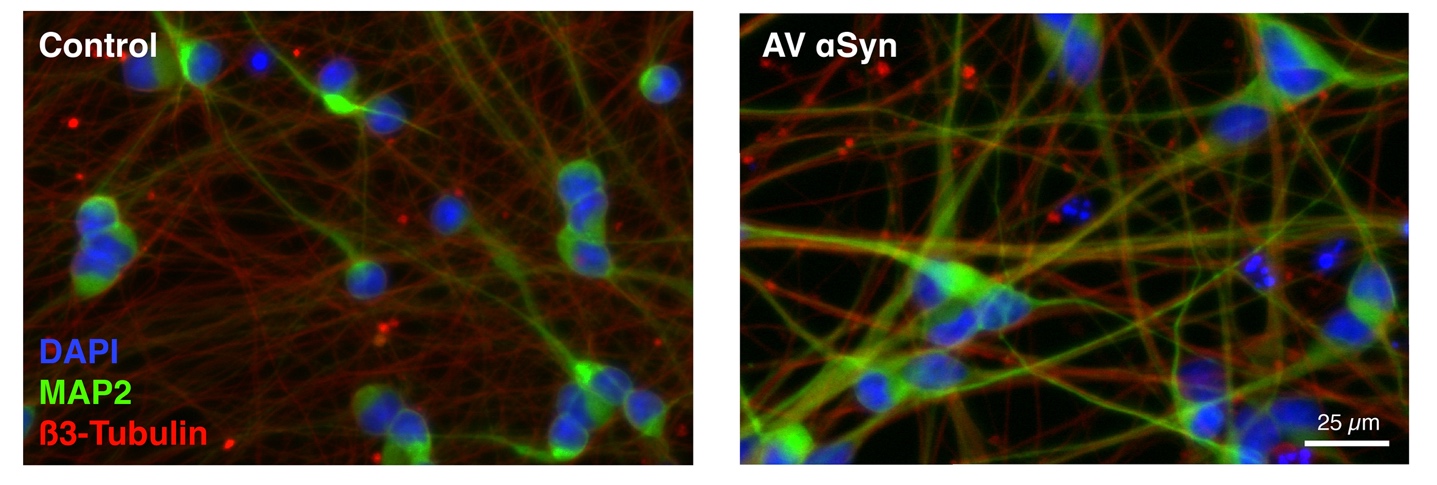


**Supplementary Figure 2.** Immunocytochemistry staining of non-transduced and αSyn-transduced cells at DIV8 (day 6 post transduction) with anti-MAP2 and anti-beta-III-tubulin. Similar intensities of MAP2 and beta-III-tubulin, and comparable neuronal morphological phenotypes can be seen in both, control and αSyn-overexpressing cells.


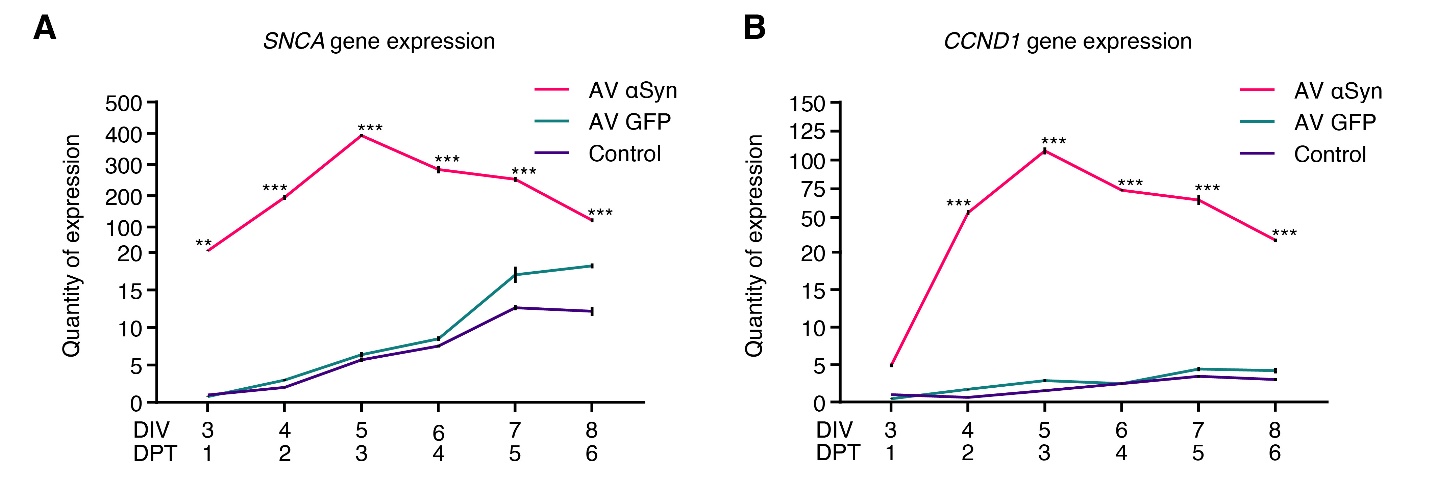


**Supplementary Figure 3.** Relative quantification of gene expression over time. Gene expression of *SNCA* **(A)** and *CCND1* **(B)** measured over time by qRT-PCR in LUHMES neurons transduced with adenoviral (AV) vectors of SNCA or GFP (control virus) or non-transduced (control). Values are related to non-transduced control at DIV 3 (DPT1). Values in (A, B) are mean ± SEM from at least 3 biological repeats. Statistical analysis was ordinary two-way ANOVA with Sidak’s multiple comparison test and, if signiﬁcant, pairwise comparisons were evaluated by an unpaired *t*-test. **P < 0.01, ***P < 0.001 vs. non-transduced control in (A, B); DIV, days *in vitro*; DPT, days post transduction.


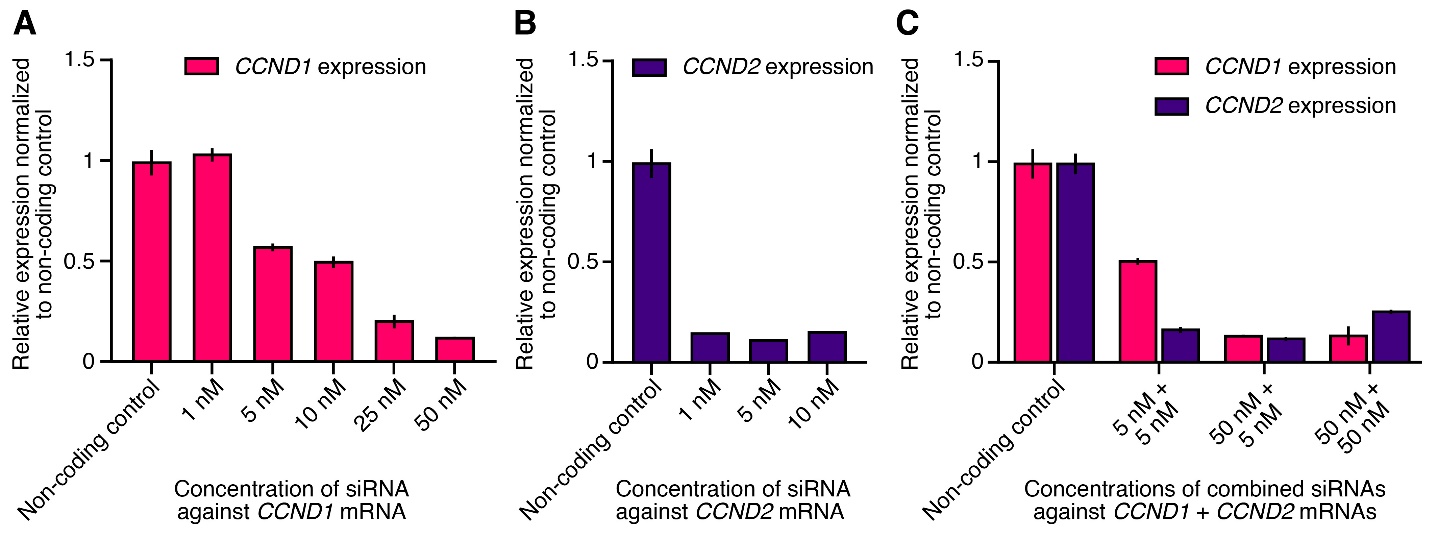


**Supplementary Figure 4.** Silencing efficiency of small interfering (si) RNA treatment. Gene expression of *CCND1* and *CCND2* in *SNCA*-overexpressing LUHMES neurons was measured by qRT-PCR on DIV6 (DPT4) upon treatment with different concentrations of siRNAs against *CCND1* (**A**), *CCND2* (**B**) or both in combination (**C**). Values are related to non-coding control. Values in (A - C) are mean ± SEM from at least 3 biological repeats. DIV, days *in vitro*; DPT, days post transduction.

# Supplementary Tables

**Supplementary Table 1.** Significantly altered miRNAs (P < 0.01) revealed by miRNome-wide screening and references linked to synucleinopathies.

| **miRNA** | **Log2 Fold Change** | **P-value** | **P_adj_-value** | **Previous Reference to Synucleinopathies** |
| --- | --- | --- | --- | --- |
| hsa-miR-663a | -1.08 | 0.0005 | 0.0644 | - |
| hsa-miR-6841-5p | -0.78 | 0.0034 | 0.0644 | - |
| hsa-miR-320c-1 | -0.76 | 0.0025 | 0.0694 | Briggs et al., 2015 |
| hsa-miR-320d-1 | -0.74 | 0.0086 | 0.0901 | Briggs et al., 2015 |
| hsa-miR-320c-2 | -0.73 | 0.0057 | 0.0901 | Briggs et al., 2015 |
| hsa-miR-1224-5p | -0.72 | 0.0011 | 0.1402 | Hoss et al., 2016; Sibley et al., 2012; |
| hsa-miR-887-3p | -0.69 | 0.0087 | 0.1402 | Hoss et al., 2016 |
| hsa-miR-1246 | -0.68 | 0.0033 | 0.1402 | - |
| hsa-miR-664a-5p | -0.59 | 0.0021 | 0.1803 | - |
| hsa-let-7e-5p | -0.51 | 0.0083 | 0.1803 | Prajapati et al., 2015; Shamsuzzama et al., 2017 |
| hsa-miR-92b-3p | -0.51 | 0.0076 | 0.1803 | Soreq et al., 2013 |
| hsa-miR-758-3p | 0.52 | 0.0044 | 0.1803 | - |
| hsa-miR-1273h-5p | 0.57 | 0.0070 | 0.1803 | - |
| hsa-miR-3929 | 0.60 | 0.0006 | 0.1803 | - |
| hsa-miR-1273e | 0.60 | 0.0031 | 0.1803 | - |
| hsa-miR-2682-5p | 0.60 | 0.0014 | 0.2199 | - |
| hsa-miR-1273a | 0.62 | 0.0027 | 0.2677 | - |
| hsa-miR-34c-5p | 0.66 | 0.0077 | 0.3022 | Kabaria et al., 2015; Minones-Moyano et al., 2011 |
| hsa-miR-143-3p | 0.70 | 0.0002 | 0.3022 | - |
| hsa-miR-27a-5p | 0.71 | 0.0013 | 0.3022 | Briggs et al., 2015; |
| hsa-miR-34a-5p | 0.78 | 0.0030 | 0.3022 | Kabaria et al., 2015 |
| hsa-miR-184 | 0.94 | 0.0001 | 0.3022 | Briggs et al., 2015; |
| hsa-miR-4483 | 1.09 | 0.0003 | 0.3022 | - |

**Supplementary Table 2.** Biological processes discovered by PANTHER GO-slim analysis.

|  |  | ***Homo sapiens***  **(Reference)** | **154 miRNA Target Genes**  **(Input)** | | |  |  |
| --- | --- | --- | --- | --- | --- | --- | --- |
| **PANTHER GO-slim Biological Process** | **Class ID** | **#** | **#** | **Expected** | **FE** | **FDR** | **P value** |
| Regulation of cyclin-dependent protein serine/threonine kinase activity | GO:0000079 | 43 | 4 | 0.32 | 12.44 | 3.17E-02 | 4.06E-04 |
| - Regulation of cell cycle | GO:0051726 | 124 | 7 | 0.93 | 7.55 | 8.39E-03 | 5.61E-05 |
| - Regulation of cellular process | GO:0050794 | 1126 | 23 | 8.42 | 2.73 | 2.72E-03 | 1.36E-05 |
| - Regulation of biological process | GO:0050789 | 2673 | 47 | 19.99 | 2.35 | 2.02E-05 | 2.25E-08 |
| - Biological regulation | GO:0065007 | 3089 | 50 | 23.1 | 2.16 | 3.36E-05 | 9.36E-08 |
| - Regulation of metabolic process | GO:0019222 | 1347 | 28 | 10.07 | 2.78 | 2.92E-04 | 9.77E-07 |
| - Regulation of cellular metabolic process | GO:0031323 | 473 | 14 | 3.54 | 3.96 | 3.04E-03 | 1.69E-05 |
| - Regulation of phosphorylation | GO:0042325 | 219 | 9 | 1.64 | 5.5 | 8.71E-03 | 5.33E-05 |
| - Regulation of phosphate metabolic process | GO:0019220 | 250 | 13 | 1.87 | 6.95 | 3.92E-05 | 8.74E-08 |
| - Regulation of phosphorus metabolic process | GO:0051174 | 250 | 13 | 1.87 | 6.95 | 5.23E-05 | 8.74E-08 |
| Regulation of cell proliferation | GO:0042127 | 96 | 6 | 0.72 | 8.36 | 1.46E-02 | 1.14E-04 |
| Cell proliferation | GO:0008283 | 82 | 5 | 0.61 | 8.15 | 3.58E-02 | 4.78E-04 |
| Positive regulation of signal transduction | GO:0009967 | 112 | 6 | 0.84 | 7.16 | 2.39E-02 | 2.53E-04 |
| - Positive regulation of response to stimulus | GO:0048584 | 117 | 6 | 0.87 | 6.86 | 2.58E-02 | 3.16E-04 |
| - Positive regulation of biological process | GO:0048518 | 254 | 9 | 1.9 | 4.74 | 1.89E-02 | 1.58E-04 |
| Protein phosphorylation | GO:0006468 | 393 | 13 | 2.94 | 4.42 | 2.49E-03 | 1.11E-05 |
| - Cellular process | GO:0009987 | 6070 | 81 | 45.39 | 1.78 | 5.34E-06 | 2.97E-09 |
| Apoptotic process | GO:0006915 | 321 | 10 | 2.4 | 4.17 | 2.12E-02 | 1.89E-04 |
| - Programmed cell death | GO:0012501 | 327 | 10 | 2.45 | 4.09 | 2.31E-02 | 2.18E-04 |
| - Cell death | GO:0008219 | 342 | 10 | 2.56 | 3.91 | 2.64E-02 | 3.09E-04 |
| Cell cycle | GO:0007049 | 605 | 13 | 4.52 | 2.87 | 4.65E-02 | 7.25E-04 |
| Intracellular signal transduction | GO:0035556 | 777 | 16 | 5.81 | 2.75 | 2.53E-02 | 2.81E-04 |
| - Signal transduction | GO:0007165 | 1685 | 26 | 12.6 | 2.06 | 3.61E-02 | 5.43E-04 |
| - Cellular response to stimulus | GO:0051716 | 1977 | 31 | 14.78 | 2.1 | 1.22E-02 | 8.81E-05 |
| Regulation of transcription, DNA-templated | GO:0006355 | 687 | 14 | 5.14 | 2.73 | 4.67E-02 | 7.53E-04 |
| - Regulation of nucleic acid-templated transcription | GO:1903506 | 687 | 14 | 5.14 | 2.73 | 4.51E-02 | 7.53E-04 |
| Transcription by RNA polymerase II | GO:0006366 | 1011 | 19 | 7.56 | 2.51 | 2.34E-02 | 2.34E-04 |
| - Transcription, DNA-templated | GO:0006351 | 1251 | 21 | 9.35 | 2.24 | 3.72E-02 | 5.39E-04 |
| - Nucleobase-containing compound metabolic process | GO:0006139 | 83 | 5 | 0.62 | 8.06 | 3.62E-02 | 5.04E-04 |

FE, fold enrichment; FDR, Benjamini-Hochberg False Discovery Rate

**Supplementary Table 3.** Primer sequences.

| **Reference Gene** |  | **Primer Pair Sequence** | |
| --- | --- | --- | --- |
|  |  | **Forward** | **Reverse** |
| *ACTB* | Actin beta | TCACCAACTGGGACGACATG | GAGGCGTACAGGGATAGCAC |
| *CCND1* | G1/S-specific cyclin-D1 | GCTGCGAAGTGGAAACCATC | CCTCCTTCTGCACACATTTGAA |
| *CCND2* | G1/S-specific cyclin-D2 | CTGTCTCTGATCCGCAAGCAT | GGTGGGTACATGGCAAACTTAAA |
| *CCNE2* | G1/S-specific cyclin-E2 | TCAAGACGAAGTAGCCGTTTAC | TGACATCCTGGGTAGTTTTCCTC |
| *CDKN1A* | Cyclin-dependent kinase inhibitor 1A (P21, Cip1) | CGATGGAACTTCGACTTTGTCA | GCACAAGGGTACAAGACAGTG |
| *CDKN1C* | Cyclin-dependent kinase inhibitor 1C (P57, Kip2) | GCGGCGATCAAGAAGCTGT | GCTTGGCGAAGAAATCGGAGA |
| *CDK6* | Cyclin dependent kinase 6 | GCTGACCAGCAGTACGAATG | GCACACATCAAACAACCTGACC |
| *CDK4* | Cyclin dependent kinase 4 | TCAGCACAGTTCGTGAGGTG | GTCCATCAGCCGGACAACAT |
| *E2F3* | E2F transcription factor 3 | AGAAAGCGGTCATCAGTACCT | TGGACTTCGTAGTGCAGCTCT |
| *E2F5* | E2F transcription factor 5 | ATGTCTTCTGACGTGTTTCCTC | CGGGGTAGGAGAAAGCCTT |
| *GAPDH* | Glyceraldehyde-3-phosphate dehydrogenase | TCGGAGTCAACGGATTTGGT | CCTGGAAGATGGTGATGGGA |
| *GPBP1* | GC-rich promotor binding protein 1 | ATCATTCGGTCTTCAACCTTCC | ATCCTCAGTTAAGGGAGCACA |
| *RPL22* | Ribosomal protein L22 | CACGAAGGAGGAGTGACTGG | TGTGGCACACCACTGACATT |
| *SNCA* | Synuclein alpha | AAGAGGGTGTTCTCTATGTAGGC | GCTCCTCCAACATTTGTCACTT |
